# Glibenclamide and metfoRmin versus stAndard care in gEstational diabeteS (GRACES): a feasibility open label randomised trial

**DOI:** 10.1186/s12884-017-1505-3

**Published:** 2017-09-22

**Authors:** Rebecca M. Reynolds, Fiona C. Denison, Ed Juszczak, Jennifer L. Bell, Jessica Penneycard, Mark W. J. Strachan, Robert S. Lindsay, Claire I. Alexander, Corinne D. B. Love, Sonia Whyte, Fiona Mackenzie, Ben Stenson, Jane E. Norman

**Affiliations:** 1 British Heart Foundation Centre for Cardiovascular Science Queen’s Medical Research Institute, University of Edinburgh, 47 Little France Crescent, Edinburgh, EH16 4TJ UK; 2 Tommy’s Centre for Maternal and Fetal Health Research, Medical Research Council Centre for Reproductive Health, Queen’s Medical Research Institute, University of Edinburgh, 47 Little France Crescent, Edinburgh, EH16 4TJ UK; 3 National Perinatal Epidemiology Unit Clinical Trials Unit, Nuffield Department of Population Health, University of Oxford, Oxford, OX3 7LF UK; 4 Metabolic Unit, Western General Hospital, Edinburgh, EH4 2XU UK; 5 Institute of Cardiovascular and Medical Sciences, University of Glasgow, Glasgow, G12 8TA UK; 6 Simpson Centre for Reproductive Health, Royal Infirmary of Edinburgh, Edinburgh, EH16 4TJ UK; 7 Neonatal Unit, Simpson Centre for Reproductive Health, Royal Infirmary of Edinburgh, Edinburgh, EH16 4TJ UK

**Keywords:** Gestational diabetes, Metformin, Glibenclamide, Feasibility, Patient preference

## Abstract

**Background:**

Metformin is widely used to treat gestational diabetes (GDM), but many women remain hyperglycaemic and require additional therapy. We aimed to determine recruitment rate and participant throughput in a randomised trial of glibenclamide compared with standard therapy insulin (added to maximum tolerated metformin) for treatment of GDM.

**Methods:**

We conducted an open label feasibility study in 5 UK antenatal clinics among pregnant women 16 to 36 weeks’ gestation with metformin-treated GDM. Women failing to achieve adequate glycaemic control on metformin monotherapy were randomised to additional glibenclamide or insulin. The primary outcome was recruitment rate. We explored feasibility with uptake, retention, adherence, safety, glycaemic control, participant satisfaction and clinical outcomes.

**Results:**

Records of 197 women were screened and 23 women randomised to metformin and glibenclamide (*n* = 13) or metformin and insulin (*n* = 10). Mean (SD) recruitment rate was 0.39 (0.62) women/centre/month. 9/13 (69.2%, 95%CI 38.6–90.9%) women adhered to glibenclamide and all provided outcome data (100% retention). There were no episodes of severe hypoglycaemia, but metformin and insulin gave superior glycaemic control to metformin and glibenclamide, with fewer blood glucose readings <3.5 mmol/l (median [IQR] difference/woman/week of treatment 0.58 [0.03–1.87]).

**Conclusions:**

A large randomised controlled trial comparing glibenclamide or insulin in combination with metformin for women with GDM would be feasible but is unlikely to be worthwhile, given the poorer glycaemic control with glibenclamide and metformin in this pilot study. The combination of metformin and glibenclamide should be reserved for women with GDM with true needle phobia or inability to use insulin therapy.

**Trial registration:**

www.clinicaltrials.gov registration number:NCT02080377 February 11th 2014.

**Electronic supplementary material:**

The online version of this article (10.1186/s12884-017-1505-3) contains supplementary material, which is available to authorized users.

## Background

Gestational diabetes mellitus (GDM), defined as carbohydrate intolerance of variable severity with onset or first recognition during pregnancy [1], is associated with increased risks of perinatal mortality and morbidity. Glucose lowering treatment in women with GDM improves outcomes including reducing birth weight, the proportion of large for gestational age infants, caesarean section and perinatal morbidity [2, 3]. Lowering diagnostic thresholds [1, 4, 5], and an increasing prevalence of risk factors such as obesity have led to many more women being diagnosed with GDM and requiring glucose lowering treatment.

Insulin has traditionally been the first drug of choice for treatment of GDM if diet and lifestyle advice fail to lower glucose adequately. Oral anti-diabetic agents including metformin (a biguanide) and glibenclamide (glyburide, a sulphonylurea) are increasingly considered attractive alternatives to insulin with equivalent efficacy to insulin, lower cost, ease of administration and patient preference [6–9]. Metformin is now used as first-line therapy in the UK, although both oral agents are endorsed in national guidelines [1, 4]. In the USA, glibenclamide is more widely used first-line with prescriptions increasing from 7.4% in 2001 to 64.5% in 2011 [10]. Nevertheless, with reported “failure rates” of metformin in three randomised studies between 32% and 46% [6, 11, 12], and of glibenclamide between 16% and 25% [13–16], a significant number of women require supplementary or alternative treatment with insulin.

Combination treatment with sulphonylureas and metformin is well established for treatment of type 2 diabetes in non-pregnancy [17–23] but has not (to our knowledge) been tested in pregnancy [20–23]. Combination therapy may be a desirable approach for women with GDM with glucose levels remaining above the target range despite maximum tolerated oral monotherapy. Such a regimen has the potential to avoid the discomfort of subcutaneous injections and the expense of insulin therapy. However, it is not known whether using the combination therapy in pregnancy is associated with increased risk of hypoglycaemia or side effects, though neither are more common with combination therapy in non-pregnancy [20–23].

In women with GDM who failed to achieve adequate glycaemic control on metformin monotherapy, we hypothesised that combination therapy with glibenclamide, compared to insulin would result in similar glycaemic control and clinical outcomes, and would be preferable to women. In this feasibility study, we aimed to determine the recruitment rate/participant throughput in a randomised controlled trial of glibenclamide compared with insulin (in addition to maximum tolerated metformin), for the treatment of GDM and to explore glycaemic control and compare clinical outcomes.

## Methods

### Study design and participants

We conducted an open-label feasibility study between 1st August 2014 and 31st October 2015 in antenatal clinics at 5 NHS hospitals in Scotland, UK. Pregnant women were eligible to participate if they had GDM (diagnosed using the definitions used at the recruiting sites which are based on the International Association of the Diabetes and Pregnancy Study Groups (IADPSG) criteria of fasting plasma glucose ≥5.1 mmol/L or 2 h plasma glucose during 75 g oral glucose tolerance test ≥8.5 mmol/L) and were failing to achieve adequate glycaemic control despite maximum tolerated dose of metformin, and were ≥16 weeks’ or ≤36 weeks’ gestation. Inadequate glycaemic control was defined according to criteria used in the Australian Carbohydrate Intolerance Study in Pregnant women (ACHOIS) study [1] and adopted in the relevant national guideline^1^ as more than two home blood glucose readings during a fortnight of any of fasting ≥5.5 mmol/L, 2 h post prandial ≥7 mmol/L, or a post prandial value at any time ≥ 9 mmol/L. We excluded women taking metformin dose <500 mg/ day; women with suspected type 1 diabetes presenting in pregnancy or marked hyperglycaemia (fasting ≥7 mmol/L, 2 h ≥ 11.1 mmol/L); women with allergies to either glibenclamide, insulin or any of their excipients; women with contraindications to sulphonylurea therapy; and women unable to give informed consent.

The study was approved by the Scotland A Research Ethics Committee (reference number:13/SS/0223) and the Medicines and Healthcare products Regulatory Agency (EudraCT number:2013–004706-25) and was registered with clinical trials.gov (registration number:NCT02080377). The trial was overseen by a Trial Steering Committee and an independent Data Monitoring Committee.

### Randomisation and masking

We randomly assigned women to receive glibenclamide or insulin (standard care) in addition to their metformin therapy using a secure web-based randomisation system hosted by the NPEU Clinical Trials Unit, University of Oxford. Randomisation used a minimisation algorithm to ensure allocation concealment, target a 1:1 allocation ratio and balance between the groups with respect to study site, BMI status (BMI <40 or ≥40 kg/m^2^) recorded at antenatal booking (typically between 11 and 13 weeks’ gestation) and multiplicity (singleton or multiple pregnancy). Neither participants nor caregivers/those collecting outcome data were masked to treatment allocation; however, using minimisation made it impossible to predict the next allocation with any certainty.

### Procedures

Participants were given verbal and written information about the study at the time of diagnosis of GDM. Prior to recruitment, treatment with metformin was commenced if women were failing to achieve adequate glycaemic control with lifestyle measures alone, according to standard practice [1, 4]. Women were commenced on metformin 500 mg once daily and the dose up-titrated as tolerated to a maximum of 2 g daily in two divided doses. If it became clear that metformin therapy was insufficient to maintain normoglycaemia, women were given further information about the study. Those wishing to participate gave written consent and were randomised. Demographics, medical history, gestation of diagnosis of GDM and dose of metformin therapy were recorded at the time of randomisation.

Participants randomised to glibenclamide were prescribed glibenclamide 2.5 mg once daily (time of day decided by their clinician according to home blood glucose monitoring) in addition to their current dose of metformin. Glibenclamide therapy was up-titrated to a maximum dose of 20 mg daily in two or three divided doses during clinic visits by diabetes specialist nurses and/or research midwives with diabetes/obstetric medical support, according to a treatment algorithm drawn up by consensus amongst study clinicians at the start of the trial (RMR, MWJS, RL) (Additional file 1: Fig. S1). All participating prescribers received training in use of the algorithm. Women were phoned three to five days after starting glibenclamide or after any dose increase in glibenclamide, with up-titration of treatment if sustained hyperglycaemia occurred. Similarly, if hypoglycaemia occurred, the dose was down-titrated. If women failed to achieve adequate glycaemic control with dual oral therapy, glibenclamide was discontinued and replaced with insulin therapy. Women randomised to insulin (standard care) were treated according to the clinician’s standard practice and doses adjusted to achieve target glycaemic control. For those who achieved normoglycaemia, allocation to glibenclamide or insulin treatment, in combination with the maximum tolerated dose of metformin, was maintained until delivery.

Participants were asked to check their blood glucose 4 times per day (fasting prior to breakfast, and 2 h post-breakfast, lunch and evening meal). Data on the participants’ blood glucose readings were directly downloaded during each clinic visit from the home blood glucose meter using the Diasend® platform (Diasend Ltd., London, UK) into a Microsoft Excel spreadsheet. We assumed that the first blood sample of the day was a “fasting” sample, and that where multiple readings of glucose were available within five minutes of each other, the last of the series was the correct reading. Clinical information was extracted from the clinical record and details of daily drug treatment were extracted from the participants’ treatment diaries at each clinic visit. Clinical outcomes including gestational weight gain, birthweight, gestation at delivery, mode of delivery, and any complications including neonatal hypoglycaemia were collected from the clinical record post-delivery. Women were asked to complete a questionnaire using a visual analogue scale to assess satisfaction at 38–40 weeks gestation and to answer the question ‘If you were given the choice in the future, would you prefer to receive insulin injections, or the glibenclamide tablets if you had diabetes again?’

### Outcomes

The primary outcome was the absolute number and throughput/recruitment rate of eligible women with GDM who were on maximum tolerated metformin and who agreed to be randomised to glibenclamide or insulin. Other key feasibility outcomes included: the proportion of women who agreed to participate (uptake); the proportion of women randomised who were retained in the study (retention); the proportion of women who remained on allocated treatment (adherence) and provided outcome data (i.e. who were not lost to follow-up); the proportion of clinicians who adhered to the protocol per se (compliance); the safety of the two treatment regimens including the number of episodes of hypoglycaemia needing treatment; any other serious adverse events (SAEs, defined as an untoward medical occurrence that does not necessarily have a causal relationship with the investigational medicinal product but is life-threatening, requires hospitalisation, consists of a birth defect or congenital abnormality, results in death, persistent or significant incapacity or disability, or any other serious medical event), and suspected unexpected serious adverse reactions (SUSARs, defined as any adverse event that is serious and suspected to be causally related to the investigational medicinal product).

Secondary outcomes were a) glycaemic control using glucose values from downloaded home blood glucose monitoring including number and percentage of excursions in blood glucose below 3.5 mmol/L (defined in this study as ‘hypoglycaemia’ [2]), and number and percentage of excursions in blood glucose above or equal to 7.0 mmol/L at the 2 h post-prandial test, and above or equal to 5.5 mmol/L at fasting test; b) participant satisfaction; c) clinical outcomes including change in maternal weight between booking and 36 weeks’ gestation, mode and gestation of delivery, birthweight z-score (adjusted for sex and gestation at birth), incidence of neonatal hypoglycaemia (defined as any of the following: blood glucose <2.6 mmol/L in first 48 h of age, or given intravenous glucose or any other drug to increase blood glucose); d) other components of the primary outcome used in the Metformin in Gestational Diabetes (MIG) study [6] - Apgar less than 7, need for phototherapy, respiratory distress syndrome (need for at least 4 h of respiratory support with supplemental oxygen, continuous positive airway pressure, or intermittent positive-pressure ventilation during the first 24 h after delivery), birth trauma (injury to the baby at delivery, defined as mild if bruises or abrasions were present at birth but resolved before 6 weeks post-partum; or moderate or serious for other injuries including fractures, Erb’s palsy and brachial plexus injuries).

### Statistical analysis

We aimed to randomise at least 22 women to each arm (total *n* = 44). As this was a feasibility study a formal power calculation was not considered appropriate [24].

Analysis was conducted in the intention-to-treat population. Maternal and infant demographic and clinical characteristics were described at baseline.

For analysis of the primary outcome, the mean and standard deviation are presented with 95% confidence intervals for the monthly recruitment rate, overall and by centre, and the overall recruitment rate/centre/month, assuming a Poisson distribution. For the other feasibility metrics (uptake rate, retention, adherence), counts and percentages were calculated (by trial arm, where appropriate) with overall 95% confidence intervals (CI). Safety outcomes are presented as counts and percentages of occurrences, by trial arm.

Unadjusted comparative analyses were performed for clinical outcomes, except where the number of events in either arm was less than 2, in which case overall counts with 95% CIs were given. For continuous outcomes, results are given as differences in means, or differences in medians (if the data were skewed) with 95% CIs. For binary or categorical outcomes, risk ratios with corresponding CIs were calculated. Participant satisfaction was presented as counts and percentages by trial arm, and overall 95% CIs. Blood glucose outcomes (waking and post-prandial) were compared by trial arm. To adjust for variation in the duration of therapy in the groups, prior to unblinding it was agreed to determine number of excursions per woman, per week of treatment, and to present the number and percentage of women with at least one excursion. Some post-hoc exploratory comparisons were made on the blood glucose readings including on the number of blood glucose readings, and the number of readings per woman per day; the overall variation in the readings. Excursions in blood glucose were compared using the number of women with at least one excursion by trial arm, with risk ratios and 95% CIs. The number of excursions/woman/week was also summarised using medians (IQR), and compared by trial arm with the median difference and 95% CI.

Stata/SE for Windows (version 13.1) was used for all analyses.

## Results

**Fig. 1:**
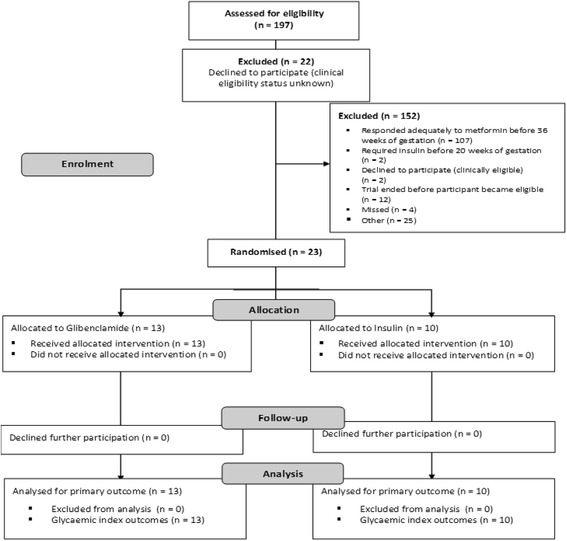
CONSORT flow chart

We screened 197 pregnant women with GDM who were taking metformin, 25 of whom satisfied the clinical eligibility criteria (CONSORT flow chart, Fig. 1). Of these, 23 women (92%, 95%CI 74–99%) agreed to participate in the trial, equivalent to a mean recruitment rate (SD) of 0.39 (0.62) women/centre/month. The most common reason for non-participation was that satisfactory glycaemic control was achieved with metformin monotherapy prior to 36 weeks’ gestation, with 107/174 (61%) of women expressing initial interest in the study being effectively treated with metformin monotherapy. The 23 participants were randomly assigned to glibenclamide (*n* = 13) or insulin (*n* = 10) in addition to metformin therapy, and all were included in the analyses of primary and secondary outcomes, i.e. no post-randomisation exclusions and 100% retention (Fig. 1). Adherence to the glibenclamide intervention was achieved in 9/13 (69.2%, 95%CI 38.6–90.9%) women, with 4 women switched to insulin therapy due to hyperglycaemia (*n* = 2) or hypoglycaemia (n = 2).

Baseline demographics and dose of metformin at recruitment to the study were broadly similar between groups, although women randomised to glibenclamide tended to be diagnosed with GDM earlier in pregnancy, and to be of higher body mass index (Table 1).

**Table 1:** Maternal characteristics at baseline (*n* = 23)

		Intervention (Glibenclamide)(*n* = 13)		Standard (Insulin) (*n* = 10)		Total (*n* = 23)	
Maternal age (years)	Mean (SD)	33.0 (5.1)		34.5 (4.9)		33.7 (5.0)	
BMI (kg/m^2^) at randomisation							
< 40	n (%)	8 (61.5)		8 (80.0)		16 (69.6)	
≥ 40	n (%)	5 (38.5)		2 (20.0)		7 (30.4)	
	Median [IQR]	39.2 [33.2 to 40.6]		33.3 [29.5 to 38.2]		35.7 [32.0 to 40.5]	
Gestational age at randomisation (weeks)	Mean (SD)	29.6 (6.3)		31.5 (2.2)		30.4 (4.9)	
Gestation at diagnosis of gestational diabetes (weeks)	Median [IQR]	24.9 [17.6 to 28.9]		27.1[24.0 to 28.9]		26.7[17.6 to 28.9]	
Daily dose of metformin at randomisation (mg)	Median [IQR]	1500 [1500 to 2000]		1500 [1500 to 2000]		1500 [1500 to 2000]	
Ethnicity							
White	n (%)	10	(76.9)	7	(70.0)	17	(73.9)
Indian	n (%)	0	(0.0)	2	(20.0)	2	(8.7)
Pakistani	n (%)	1	(7.7)	1	(10.0)	2	(8.7)
Bangladeshi	n (%)	0	(0.0)	0	(0.0)	0	(0.0)
Other Asian background	n (%)	1	(7.7)	0	(0.0)	1	(4.4)
Black	n (%)	1	(7.7)	0	(0.0)	1	(4.4)
Deprivation level^a^							
1 (Most deprived)	n (%)	6	(46.2)	4	(40.0)	10	(43.5)
2	n (%)	3	(23.1)	2	(20.0)	5	(21.7)
3	n (%)	1	(7.7)	0	(0.0)	1	(4.3)
4	n (%)	2	(15.4)	1	(10.0)	3	(13.0)
5 (Least deprived)	n (%)	1	(7.7)	3	(30.0)	4	(17.4)
Number of previous pregnancies^b^							
0	n (%)	1	(7.7)	2	(22.2)	3	(13.6)
1 or more	n (%)	12	(92.3)	7	(77.8)	19	(86.4)
Blood pressure (mmHg)							
Systolic	Mean (SD)	118.4	(11.4)	122.2	(10.8)	120.0	(11.0)
Diastolic	Mean (SD)	72.2	(7.0)	72.6	(8.7)	72.3	(7.6)

^a^ From the Scottish Index of Multiple Deprivation http://www.isdscotland.org/Products-and-Services/GPD-Support/Deprivation/SIMD/index.asp?Co=Y

^b^ All lengths, including miscarriages; data missing from 1 participant in the insulin-treatment group

**Fig. 2:**
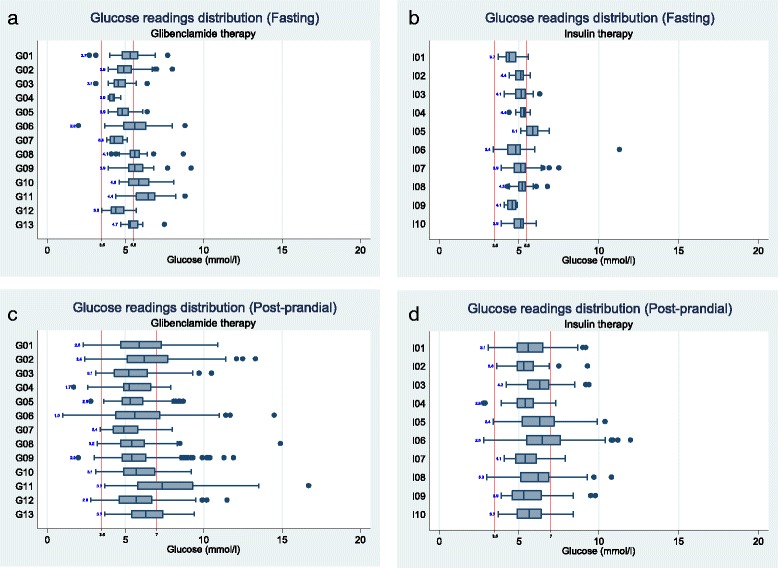
Fasting and post-prandial home blood glucose readings in women with GDM treated with metformin and randomised to glibenclamide (G1-13 Panels **a** and **c**) or insulin (I 1-10 Panels **b** and **d**)

Our pre-specified primary safety outcome was hypoglycaemia requiring assistance – none of the women in either group experienced this or any other SUSAR (Table 2). There were four women with a serious adverse event - three with a post-partum haemorrhage and one with sepsis, but these were equally distributed between the groups (Table 2). Women treated with glibenclamide had significantly more episodes of asymptomatic hypoglycaemia per week (median difference (IQR) of number of excursions <3.5 mmol/L per woman per week 0.58 (0.03 to 1.87)) (Additional file 2: Table S1) and tended to have more fasting excursions ≥5.5 mmol/L (median difference (IQR) of number of excursions per woman per week 0.50 (−0.55 to 2.33)) and higher post-prandial glucose excursions (Additional file 2: Table S1 and Fig. 2).

**Table 2:** Feasibility outcomes

		Intervention (Glibenclamide) (n = 13)		Standard (Insulin) (n = 10)		Total (n = 23)(95% CI)	
Primary outcome: Number of women who agreed to be randomised							
Overall monthly recruitment rate (15.8 months) ^a^	Mean {SD}					1.45	{1.21}
						(0.92 to 2.18)	
Recruitment rate per centre per month (58.9 months) ^b^	Mean {SD}					0.39	{0.62}
						(0.25 to 0.59)	
Uptake rate							
Total number of women eligible	n					25	
Proportion of those eligible^c^ agreeing to be randomised	n (%)					23	(92.0)
						(74.0% to 99.0%)	
Retention^d^							
Number of women who remained in the study	n (%)	13	(100.0)	10	(100.0)	23	(100.0)
						(85.2% to 100.0%)	
Adherence **-** Adhered to the treatment regimen^e^	n (%)	9^f^	(69.2)				
	(95% CI)	(38.6% to 90.9%)					
Safety^g^							
Number of women with hypoglycaemic episodes needing treatment	n (%)	0 (0.0)		0 (0.0)		0 (0.0)	
Number of women with SUSARs	n (%)	0 (0.0)		0 (0.0)		0 (0.0)	
Number of women with SAEs	n (%)	2 (15.4)		2 (20.0)		4 (17.4)	
Number of women with other adverse events	n(%)	0 (0.0)		0 (0.0)		0 (0.0)	

^a^ Numerator is the number of women randomised, denominator is the total length the trial was recruiting (15.8 months)

^b^ Numerator is the number of women randomised, denominator is the sum of the length of time each centre was open (58.9 months)

^c^ Eligible women defined as those who were recruited or declined to participate but were known to be clinically eligible

^d^ Proportion of women who delivered and remained in the study to provide outcomes

^e^ Glibenclamide allocation only – indicates women who did not switch from Glibenclamide to insulin to maintain normoglycaemia

^f^ 4 switched for the following reasons:

2 because blood glucose measurements were high

1 not achieving adequate glycaemic control on insulin. Fasting sugars high but occasional low sugars at lunch

1 sugars too low with Glibenclamide, but too high without

^g^ As per ITT, provided that they received at least one dose of the treatment allocated

Among participants randomised to glibenclamide, the majority expressed a preference for glibenclamide therapy (Additional file 2: Table S2). However, only 1 woman (12.5% of those expressing a preference) who was randomised to insulin expressed a preference for glibenclamide, and 3 women did not complete the questionnaire (1 in glibenclamide group, 2 in insulin group). There were no significant differences between groups in any of the other secondary outcomes including gestational weight gain, birthweight, gestation at delivery, mode of delivery and other complications including neonatal hypoglycaemia, although both episodes of neonatal hypoglycaemia were in the group whose mothers were treated with glibenclamide (in addition to metformin) (Additional file 2: Table S2 and Additional file 2: Table S3).

## Discussion

### Main findings

Based on our recruitment rates of 0.39 women/centre/month, we estimate that a large randomised trial of glibenclamide compared with insulin (assuming a sample size of between 500 and 800) would require 30–60 centres recruiting for 3 years. Such a study is likely to be feasible, but expensive. However, our prior hypothesis that glibenclamide and insulin would be equivalent in terms of glycaemic and clinical outcomes appears incorrect, given that we showed significantly greater frequencies of excursions in blood glucose below 3.5 mmol/L in the glibenclamide group. Preference for oral therapy was not universal, with 45% of all participants either expressing no preference or preferring insulin.

### Interpretation

The combination of glibenclamide and metformin appears worse than the combination of insulin and metformin. Contrary to our expectations about the unattractiveness of subcutaneous injections, women did not universally express a preference for glibenclamide over insulin. Hence we believe that further trials of glibenclamide and metformin in pregnancy in unselected women with GDM are unwarranted. In this small sample, preliminary data suggest that insulin (in combination with metformin) gives superior glycaemic control, with a lower incidence of glucose excursions <3.5 mmol/L (compared with glibenclamide in combination with metformin). A recent systematic review of drug treatments for GDM highlighted an increased risk of neonatal hypoglycaemia with glibenclamide compared with insulin monotherapy [25]. The pharmacokinetics of metformin are unchanged in pregnancy, whereas oral clearance rates of glibenclamide increase [26, 27]. Whether there are further changes in pharmacokinetics of the two drugs used in combination in pregnancy is unknown. Metformin crosses the placenta, but there is debate about whether this is true for glibenclamide [26, 27]. Other factors which may have contributed to the poorer glycaemic control in the glibenclamide arm of our trial include limited flexibility to titrate doses (smallest tablet dose was 2.5 mg) and inexperience of clinicians with titrating doses due to lack of routine use of glibenclamide, which has largely been replaced by sulphonylureas with a shorter duration of action (gliclazide, glipizide) in diabetes practice in the UK. Our other secondary outcomes showed no differences between treatment with glibenclamide or insulin, but must be interpreted with caution due to the small sample size.

We were surprised to find little evidence of preference for oral therapy. A previous study found no increase in anxiety and depression symptoms in women with GDM when treatment was intensified from diet alone to insulin therapy [28]. Another study indicated the dominant concern in women with GDM was ‘the baby’s health’, whereas treatment satisfaction was generally high [29]. A woman’s concerns about choice of adjuvant therapy for GDM when metformin has failed are also likely to be different from individuals with type 2 diabetes [30] due to the much shorter duration of need for therapy.

### Strengths and limitations

The strengths of our study include the robust multicentre randomised controlled design, and the statistically significant difference in hypoglycaemic episodes in association with glibenclamide. We also demonstrate that using routinely collected clinical data including downloads of home-blood glucose measurements gives a wealth of information about maternal glycaemic control in ‘real life’. We do not believe that the open-label design impacted on outcome, as an algorithm was used by all clinicians for titration of medication doses and to guide decisions for ‘rescue’ therapy. The limitations of the study include the small sample size, which could have contributed to a chance imbalance on prognostic factors including weight and time of diagnosis. The lower than planned recruitment rate to our study occurred principally because the majority of women achieved adequate glycaemic control with metformin monotherapy, though rates of metformin ‘failure’ in around a third of women were consistent with other published studies [6, 11, 12]. A further limitation is that the lack of ethnic diversity in our sample mean our findings may not be generalisable, though we are not aware of any studies showing ethnic differences in responses to either metformin or glibenclamide monotherapy in pregnancy.

## Conclusions

In conclusion, this small feasibility study shows that a large randomised controlled trial comparing additional glibenclamide or insulin in women with GDM who are failing to achieve adequate glycaemic control with metformin alone is feasible, but is unlikely to be clinically worthwhile given both the poorer glycaemic control with glibenclamide and metformin in this pilot study and the lack of universal preference for glibenclamide over insulin. The lack of adverse effects in our study suggests that the combination of metformin and glibenclamide may be considered as treatment option for selected individual women with GDM who are failing metformin monotherapy, if there is careful monitoring of glycaemic control. Such an option may be useful for women with a needle phobia, women with an inability to use insulin therapy or where insulin is unavailable.

## Additional files

Additional file 1: Figure S1. Algorithm for glibenclamide dose adjustment. (DOCX 63 kb)

Additional file 2: Table S1. Secondary outcomes – Glycaemic control. **Table S2**. Clinical outcomes – Mother **Table S3**. Secondary outcomes – Clinical outcomes (infant). (DOCX 52 kb)

## Abbreviations

CI: Confidence Interval
GDM: gestational diabetes
IQR: interquartile range
SAE: serious adverse event
SD: standard deviation
SUSAR: suspected unexpected serious adverse reactions

## References

[1] Scottish Intercollegiate Guideline Network, Management of diabetes - a national clinical guideline (no 116), 2010, NHS Quality Improvement Scotland: Edinburgh.

[2] Crowther CA, Hiller JE, Moss JR, McPhee AJ, Jeffries WS (2005). Robinson JS; Australian carbohydrate intolerance study in pregnant omen (ACHOIS) trial group. Effect of treatment of gestational diabetes mellitus on pregnancy outcomes. NEJM.

[3] Landon MB, Spong CY, Thom E, Carpenter MW, Ramin SM, Casey B (2009). A multicentre, randomized trial of treatment for mild gestational diabetes. NEJM.

[4] National Institute for Clinical Excellence. Diabetes in Pregnancy: Management from pre-conception to the postnatal period. NICE Guidelines [NG3] Published February 2015.

[5] Metzger BE, Gabbe SG, Persson B, Buchanan TA, Catalano PA, Damm P (2010). International association of diabetes and pregnancy study groups recommendations on the diagnosis and classification of hyperglycaemia in pregnancy. Diab Care.

[6] Rowan JA, Hague WM, Gao W, Battin MR, Moore MP (2008). MiG Trial Investigators. Metformin versus insulin for the treatment of gestational diabetes. NEJM.

[7] Langer O, Conway DL, Berkus MD, Xenakis EM, Gonzales O (2000). A comparison of glyburide and insulin in women with gestational diabetes mellitus. NEJM.

[8] Dhulkotia JS, Ola B, Fraser R, Farrell T (2010). Oral hypoglycemic agents vs insulin in management of gestational diabetes: a systematic review and metaanalysis. Am J Obstet Gynecol.

[9] Alwan N, Tuffnell DJ, West J (2009). Treatments for gestational diabetes. Cochrane Database Syst Rev.

[10] Camelo Castillo W, Boggess K, Sturmer T, Brookhart MA, Benjamin DK, Jonsson FM (2014). Trends in glyburide compared with insulin use for gestational diabetes treatment in the United States, 2000-2011. Obstet Gynecol.

[11] Moore LE, Clokey D, Rappaport VJ, Curet LB (2010). Metformin compared with glyburide in gestational diabetes: a randomized controlled trial. Obstet Gynecol.

[12] Ijas H, Vääräsmäki M, Morin-Papunen L (2011). Metformin should be considered in the treatment of gestational diabetes: a prospective randomized study. BJOG.

[13] Harper LM, Glover AV, Biggio JR, Tita A. Predicting failure of glyburide therapy in gestational diabetes. J Perinatol. 2016 Jan 21; 10.1038/jp.2015.216. [Epub ahead of print] 10.1038/jp.2015.216 PMC4844846 26796130

[14] Yogev Y, Melamed N, Chen R, Nassie D, Pardo J, Hod M (2011). Glyburide in gestational diabetes--prediction of treatment failure. J Mat, Fetal Neonat Med.

[15] Kahn BF, Davies JK, Lynch AM, Reynolds RM, Barbour LA (2006). Predictors of glyburide failure in the treatment of gestational diabetes. Obstet Gynecol.

[16] Conway DL, Gonzales O, Skiver D (2004). Use of glyburide for the treatment of gestational diabetes: the San Antonio experience. J Mat Fetal Neonat Med.

[17] Lamos EM, Stein SA, Davis SN (2012). Combination of glibenclamide-metformin HCL for the treatment of type 2 diabetes mellitus. Exp Op Pharmacotherapy.

[18] Inzucchi SE, Bergenstal RM, Buse JB, Diaman M, Ferrannini E, Nauck M (2012). Management of hyperglycaemia in type 2 diabetes: a patient-centreed approach: position statement of the American Diabetes Association (ADA) and the European Association for the Study of diabetes (EASD). Diab Care.

[19] National Institute for Clinical Excellence. Type 2 diabetes in adults: Management. NICE guidelines [NG28] Published December 2015.

[20] DeFronzo RA, Goodman AM (1995). Efficacy of metformin in patients with non-insulin-dependent diabetes mellitus. The Multicentre Metformin Study Group NEJM.

[21] Marre M, Howlett H, Lehert P, Allavoine T (2002). Improved glycemic control with metformin-glibenclamide combined tablet therapy (Glucovance) in type 2 diabetic patients inadequately controlled on metformin. Diab Med.

[22] Garber AJ, Donovan DS, Dandona P, Bruce S, Park JS (2003). Efficacy of glyburide/metformin tablets with initial monotherapy in type 2 diabetes. JCEM.

[23] Blonde L, Rosenstock J, Mooradian AD, Piper BA, Henry D. Glyburide/metformin combination product is safe and efficacious in patients with type 2 diabetes failing sulphonylurea therapy. Diabetes Obes Metab. 2002;4:368–75. 10.1046/j.1463-1326.2002.00229.x 12406033

[24] Whitehead AL, Julious SA, Cooper CL, Campbell MJ. Estimating the sample size for a pilot randomized trial to minimise the overall trial sample size for the external pilot and main trial for a continuous outcome variable. Stat Meth Med Res 2015; 0(0): 1–17. 10.1177/0962280215588241 PMC4876429 26092476

[25] Balsells M, García-Patterson A, Solà I, Roqué M, Gich I, Corcoy R (2015). Glibenclamide, metformin, and insulin for the treatment of gestational diabetes: a systematic review and meta-analysis. BMJ.

[26] Charles B, Norris R, Xiao X, Hague W (2006). Population pharmacokinetics of metformin in later pregnancy. Ther Drug Monit.

[27] Caritis SN, Hebert MF (2013). A pharmacologic approach to the use of glyburide in pregnancy. Obstet Gynecol.

[28] Langer N, Langer O (1994). Emotional adjustment to diagnosis and intensified treatment of gestational diabetes. Obstet Gynecol.

[29] Trutnovsky G, Panzitt T, Magnet E, Stern C, Lang U, Dorfer M (2012). Gestational diabetes: women's concerns, mood state, quality of life and treatment satisfaction. J Mat, Fet Neonat Med.

[30] Shillington AC, Col N, Bailey RA, Jewell MA (2015). Development of a patient decision aid for type 2 diabetes mellitus for patients not achieving glycemic control on metformin alone. Pat Pref Adh.

