# Supplementary material for: Glibenclamide and metfoRmin versus stAndard care in gEstational diabeteS (GRACES): a feasibility open label randomised trial
Source: BMC Pregnancy Childbirth. 2017 Sep 22;17:316. doi: 10.1186/s12884-017-1505-3 (PMC5610470; doi:10.1186/s12884-017-1505-3)
Supplement: Supplementary file 1 — Algorithm for glibenclamide dose adjustment. (DOCX 63 kb) [file 12884_2017_1505_MOESM1_ESM.docx]

**GRACES Glibenclamide Suggested Dosing Guide**

Patients recruited to GRACES trial and randomised to Glibenclamide will be taking the Glibenclamide in addition to their maximum tolerated dose of metformin. The dosing schedule will have some degree of flexibility to it with regards to timing of doses and how rapidly the dose is increased, dependent upon patient glycaemic control and tolerability. Maximum dose of glibenclamide in all patients will be 20mg daily.

The decision to adjust the dose will be made by the patient’s clinician (doctor or DSN) and will be dependent upon the patient’s glycaemic control and tolerability. See dosing algorithm below.

**All patients should be contacted (either seen in clinic or via telephone consultation) between days 2-5 after commencing Glibenclamide or after a dose change, to discuss blood glucose results and make any necessary alterations to dose.**

Inadequate glycaemic control for purposes of trial:

|  | Blood glucose |
| --- | --- |
| Fasting | ≥5.5mmol/L |
| At <35 weeks gestation 2 hour post prandial | ≥7mmol/L |
| At ≥35 weeks gestation 2 hour post prandial | ≥8mmol/L |
| Or a post prandial value at any gestation | ≥9mmol/L |

**Increasing** the dose of Glibenclamide will be triggered by episodes of hyperglycaemia as defined above; the no. of episodes triggering a dose increase will be at clinician discretion.

**Reducing the dose**: Any episodes of symptomatic, unexplained HYPOglyacemia (blood glucose <3.5) should trigger the dose of glibenclamide to be down-titrated accordingly.

Triggers for **stopping** Glibenclamide treatment and reverting to standard care (metformin + insulin):

- On maximum dose Glibenclamide (20mg daily) and still not achieving adequate glucose control.
- Unable to tolerate Glibenclamide (either at any dose or at dose necessary to achieve adequate control).
- Patient request or clinician concern

**ALL DIABETES TREATMENT SHOULD STOP FOLLOWING DELIVERY**

**Algorithm for Glibenclamide dosing:**

Commence Glibenclamide 2.5mg once daily

**Tx Poorly tolerated/ hypoglycaemia:** consider reducing dose or stopping treatment and reverting to standard care.

**Subsequent clinic visits:** continue to review glycaemic control and if adjustments need to be made to dose

**In-adequate glycaemic control, maximum dose:** stop Glibenclamide and revert to standard care

**In-adequate glycaemic control, not on maximum dose:** Increase dose by 2.5 or 5mg daily to maximum of 20mg daily in divided doses. ˟

**Day 2-5 consultation (clinic or telephone) with DSN or doctor:** review glycaemic control

**Adequate glycaemic control:** continue with current dose and review in clinic in 1-2/52 or patient to contact DSN if concerns

**˟** Example of possible dosing increase regimen may be:

Day 0: 2.5mg od

Day 2: 2.5mg bd

Day 4: 2.5mg/5mg or 5mg/2.5mg

Day 6: 5mg bd

Day 8: 5mg/10mg or 10mg/5mg

Day 10: 10mg bd.
