# Supplementary material for: Glibenclamide and metfoRmin versus stAndard care in gEstational diabeteS (GRACES): a feasibility open label randomised trial
Source: BMC Pregnancy Childbirth. 2017 Sep 22;17:316. doi: 10.1186/s12884-017-1505-3 (PMC5610470; doi:10.1186/s12884-017-1505-3)
Supplement: Supplementary file 2 — Secondary outcomes – Glycaemic control. Table S2. Clinical outcomes – Mother Table S3. Secondary outcomes – Clinical outcomes (infant). (DOCX 52 kb) [file 12884_2017_1505_MOESM2_ESM.docx]

| Supplementary Table 1: Secondary outcomes – Glycaemic control | | | | | | | | | | | | | | | | | | | | | | | | | | |  | | | | |  |  |  |  |
| --- | --- | --- | --- | --- | --- | --- | --- | --- | --- | --- | --- | --- | --- | --- | --- | --- | --- | --- | --- | --- | --- | --- | --- | --- | --- | --- | --- | --- | --- | --- | --- | --- | --- | --- | --- |
|  | | |  | | | | | **Intervention (Glibenclamide) (n = 13)** | | | | | | | | | **Standard  (Insulin)  (n = 10)** | | | | | | | | | | **Effect estimate^[[1]](#footnote-1)^ (95% CI^[[2]](#footnote-2)^)** | | | | |  |  |  |  |
|  | | |  | | | | |  | | | | | | | | |  | | | | | | | | | |  | | | | |  |  |  |  |
| **Waking blood glucose**^[[3]](#footnote-3)^ **(randomisation to delivery)** | | | Mean {SD^[[4]](#footnote-4)^} | | | | | 5.2 | | | | {1.0} | | | | | 5.1 | | | | | | {0.7} | | | | MD^[[5]](#footnote-5)^ -0.15  (-0.27 to -0.04) | | | | |  |  |  |  |
|  | | |  | | | | |  | | | | | | | | |  | | | | | | | | | |  | | | | |  |  |  |  |
| **Post-prandial blood glucose**^[[6]](#footnote-6)^ **(randomisation to delivery)** | | | Mean {SD} | | | | | 5.9 | | | | {1.7} | | | | | 5.9 | | | | | | {1.2} | | | | MD -0.04 (-0.15 to 0.06) | | | | |  |  |  |  |
|  | | |  | | | | |  | | | |  | | | | |  | | | | | |  | | | |  | | | | |  |  |  |  |
| **All readings (randomisation to delivery)** | | | Mean {SD} | | | | | 5.8 | | | | {1.6} | | | | | 5.7 | | | | | | {1.2} | | | | VR^[[7]](#footnote-7)^ 0.51 (0.47 to 0.56) | | | | |  |  |  |  |
|  | | |  | | | | |  | | | | | | | | |  | | | | | | | | | |  | | | | |  |  |  |  |
| **Excursions in blood glucose** | | |  | | | | |  | | | | | | | | |  | | | | | | | | | |  | | | | |  |  |  |  |
| < 3.5 mmol/l | | |  | | | | |  | | | | | | | | |  | | | | | | | | | |  | | | | |  |  |  |  |
| Women with at least one excursion | | | n (%) | | | | | 11 | | | | (84.6) | | | | | 5 | | | | | | (50.0) | | | | RR^[[8]](#footnote-8)^ 1.69  (0.87 to 3.28) | | | | |  |  |  |  |
| Number of excursions per woman per week of treatment | | | Median [IQR^[[9]](#footnote-9)^] | | | | | 0.7 [0.2 to 1.9] | | | | | | | | | 0.1 [0.0 to 0.3] | | | | | | | | | | Med D^[[10]](#footnote-10)^ -0.58 (-1.87 to -0.03) | | | | |  |  |  |  |
| ≥ 5.5 mmol/l at fasting test | | |  | | | | |  | | | | | | | | |  | | | | | | | | | |  | | | | |  |  |  |  |
| Women with at least one excursion | | | n (%) | | | | | 11 | | | | (84.6) | | | | | 9 | | | | | | (90.0) | | | | RR 0.94  (0.69 to 1.28) | | | | |  |  |  |  |
| Number of excursions per woman per week of treatment | | | Median [IQR] | | | | | 1.3 [0.8 to 3.6] | | | | | | | | | 0.8 [0.5 to 1.3] | | | | | | | | | | Med D -0.50  (-2.33 to 0.55) | | | | |  |  |  |  |
| ≥ 7.0 mmol/l at post-prandial test | | |  | | | | |  | | | | | | | | |  | | | | | | | | | |  | | | | |  |  |  |  |
| Women with at least one excursion | | | n (%) | | | | | 13 | | | | (100.0) | | | | | 10 | | | | | | (100.0) | | | | N/A | | | | |  |  |  |  |
| Number of excursions per woman per week of treatment | | | Median [IQR] | | | | | 3.9 [1.9 to 7.5] | | | | | | | | | 3.0 [1.3 to 5.2] | | | | | | | | | | Med D -1.49  (-4.55 to 0.68) | | | | |  |  |  |  |
|  | | |  | | | | |  | | | | | | | | |  | | | | | | | | | |  | | | | |  |  |  |  |
| Supplementary Table 2 – Clinical outcomes - Mother | | | | | | | | | | | | | | | | | | | | | | | | |  | | | | | | | |  |  |  |
|  | |  | | | | **Intervention (Glibenclamide) (n = 13)** | | | | | | | | | **Standard  (Insulin)  (n = 10)** | | | | | | | | | | **Total (n = 23)  (95% CI^[[11]](#footnote-11)^)** | | | | | | | |  |  |  |
| **Participant satisfaction^[[12]](#footnote-12)^** | |  | | | |  | | | | | | | | |  | | | | | | | | | |  | | | | | | | |  |  |  |
| Prefer insulin injections | | n (%) | | | | 2 | | | | (16.7) | | | | | 1 | | | | | (12.5) | | | | | 3 | | | | | (15.0) | | |  |  |  |
|  |  |  |  |  |  |  |  |  |  |  |  |  |  |  |  |  |  |  |  |  |  |  |  |  | (3.2% to 37.9%) | | | | | | | |  |  |  |
| Prefer glibenclamide | | n (%) | | | | 10 | | | | (83.3) | | | | | 1 | | | | | (12.5) | | | | | 11 | | | | | (55.0) | | |  |  |  |
|  |  |  |  |  |  |  |  |  |  |  |  |  |  |  |  |  |  |  |  |  |  |  |  |  | (31.5% to 76.9%) | | | | | | | |  |  |  |
| No preference | | n (%) | | | | 0 | | | | (0.0) | | | | | 6 | | | | | (75.0) | | | | | 6 | | | | | (30.0) | | |  |  |  |
|  |  |  |  |  |  |  |  |  |  |  |  |  |  |  |  |  |  |  |  |  |  |  |  |  | (11.9% to 54.3%) | | | | | | | |  |  |  |
| Missing | | n | | | | 1 | | | |  | | | | | 2 | | | | |  | | | | |  | | | | | | | |  |  |  |
|  | |  | | | |  | | | | | | | | |  | | | | | | | | | |  | | | | | | | |  |  |  |
|  | | | |  | | | | |  | | | | | | | | |  | | | | | | | | | |  | | | | | | | |
| **Change in maternal weight (kg)** | | | |  | | | | |  | | | | | | | | |  | | | | | | | | | |  | | | | | | | |
| Between booking and randomisation | | | | Mean {SD^[[13]](#footnote-13)^} | | | | | 5.5 | | | | {2.7} | | | | | 5.8 | | | | | | {5.5} | | | | MD^[[14]](#footnote-14)^ 0.29  (-3.36 to 3.94) | | | | | | | |
| Between randomisation and 36 weeks (±1 week) of gestation | | | | Mean {SD} | | | | | 1.8 | | | | {3.5} | | | | | 1.0 | | | | | | {1.5} | | | | MD -0.77  (-3.55 to 2.01) | | | | | | | |
| Missing^[[15]](#footnote-15)^ | | | | n | | | | | 1 | | | |  | | | | | 2 | | | | | |  | | | |  | | | | | | | |
|  | | | |  | | | | |  | | | | | | | | |  | | | | | | | | | |  | | | | | | | |
| **Mode of delivery** | | | |  | | | | |  | | | | | | | | |  | | | | | | | | | |  | | | | | | | |
| Spontaneous vaginal delivery (SVD) | | | | n (%) | | | | | 8 | | | | (61.5) | | | | | 3 | | | | | | (30.0) | | | | RR^[[16]](#footnote-16),^^[[17]](#footnote-17)^ 0.51  (0.20 to 1.34) | | | | | | | |
| Assisted vaginal delivery | | | | n (%) | | | | | 1 | | | | (7.7) | | | | | 1 | | | | | | (10.0) | | | |  |  |  |  |  |  |  |  |
| Caesarean section – elective | | | | n (%) | | | | | 4 | | | | (30.8) | | | | | 2 | | | | | | (20.0) | | | |  |  |  |  |  |  |  |  |
| Caesarean section – emergency | | | | n (%) | | | | | 0 | | | | (0.0) | | | | | 4 | | | | | | (40.0) | | | |  |  |  |  |  |  |  |  |
|  | | | |  | | | | |  | | | | | | | | |  | | | | | | | | | |  | | | | | | | |
| **Gestation of delivery (weeks)** | | | | Median  [IQR^[[18]](#footnote-18)^] | | | | | 38.3  [38.0 to 39.4] | | | | | | | | | 38.1  [36.4 to 38.6] | | | | | | | | | | Med D^[[19]](#footnote-19)^ -0.71  (-1.86 to 0.29) | | | | | | | |
|  | | | | (Min to Max) | | | | | (37.0 to 40.3) | | | | | | | | | (34.9 to 39.1) | | | | | | | | | |  | | | | | | | |
|  | | | |  | | | | |  | | | | | | | | |  | | | | | | | | | |  | | | | | | | |
| **Delivery complications** | | | |  | | | | |  | | | | | | | | |  | | | | | | | | | |  | | | | | | | |
| Postpartum haemorrhage (PPH) | | | | n (%) | | | | | 5 | | | | (38.5) | | | | | 2 | | | | | | (22.2) | | | | RR 1.73  (0.43 to 7.04) | | | | | | | |
| Missing | | | | n | | | | | 0 | | | |  | | | | | 1 | | | | | |  | | | |  | | |  | | | | |
| Retained placenta | | | | n (%) | | | | | 0 | | | | (0.0) | | | | | 0 | | | | | | (0.0) | | | | N/A | | | | | | | |
| Missing | | | | n | | | | | 1 | | | |  | | | | | 2 | | | | | |  | | | |  | | |  | | | | |
|  | | | |  | | | | |  | | | | | | | | |  | | | | | | | | | |  | | | | | | | |
| Supplementary Table 3: Secondary outcomes – Clinical outcomes (i**n**fant) | | | | | | | | | | | | | | | | | | | | | | | | | |  | | | | | | | | |  |
|  |  | | | | **Intervention (Glibenclamide) (n = 13^[[20]](#footnote-20)^)** | | | | | | | | | **Standard  (Insulin)  (n = 10)** | | | | | | | | | | | | **Effect estimate^[[21]](#footnote-21)^ (95% CI^[[22]](#footnote-22)^)** | | | | | | | | |  |
|  |  | | | |  | | | | | | | | |  | | | | | | | | | | | |  | | | | | | | | |  |
| **Infant outcome** |  | | | |  | | | | | | | | |  | | | | | | | | | | | |  | | | | | | | | |  |
| Live birth | n (%) | | | | 13 | | | | (100.0) | | | | | 10 | | | | | (100.0) | | | | | | | N/A | | | | | | | | |  |
| Stillbirth | n (%) | | | | 0 | | | | (0.0) | | | | | 0 | | | | | (0.0) | | | | | | |  |  |  |  |  |  |  |  |  |  |
| Miscarriage^[[23]](#footnote-23)^ | n (%) | | | | 0 | | | | (0.0) | | | | | 0 | | | | | (0.0) | | | | | | |  |  |  |  |  |  |  |  |  |  |
| Perinatal death^[[24]](#footnote-24)^ | n (%) | | | | 0 | | | | (0.0) | | | | | 0 | | | | | (0.0) | | | | | | |  |  |  |  |  |  |  |  |  |  |
| Late neonatal death^[[25]](#footnote-25)^ | n (%) | | | | 0 | | | | (0.0) | | | | | 0 | | | | | (0.0) | | | | | | |  |  |  |  |  |  |  |  |  |  |
|  |  | | | |  | | | | | | | | |  | | | | | | | | | | | |  | | | | | | | | |  |
| **Babies born preterm (<37^+0^ weeks)** | n (%) | | | | 0 | | | | (0.0) | | | | | 3 | | | | | (30.0) | | | | | | | N/A | | | | | | | | |  |
|  |  | | | |  | | | | | | | | |  | | | | | | | | | | | |  | | | | | | | | |  |
| **Birth weight (g)** | Mean {SD^[[26]](#footnote-26)^} | | | | 3,706 | | | | {560} | | | | | 3,262 | | | | | | | {484} | | | | | MD^[[27]](#footnote-27)^ -444  (-907 to 19) | | | | | | | | |  |
|  |  | | | |  | | | |  | | | | |  | | | | |  | | | | | | |  | | |  | | | | | |  |
| **Birth weight z-score, adjusted for age and gestation at birth** | Mean {SD} | | | | 1.2 | | | | {1.3} | | | | | 0.6 | | | | | {0.8} | | | | | | | MD -0.54  (-1.51 to 0.44) | | | | | | | | |  |
|  |  | | | |  | | | | | | | | |  | | | | | | | | | | | |  | | | | | | | | |  |
| **Sex** |  | | | |  | | | | | | | | |  | | | | | | | | | | | |  | | | | | | | | |  |
| Male | n (%) | | | | 5 | | | | (38.5) | | | | | 5 | | | | | (50.0) | | | | | | |  | | | | | | | | |  |
|  |  | | | |  | | | | | | | | |  | | | | | | | | | | | |  | | | | | | | | |  |
|  | | | |  | | |  | | | | | | | | | | |  | | | | | | | | | |  | | | | | |  |  |
| **Delivery complications** | | | |  | | |  | | | | | | | | | | |  | | | | | | | | | |  | | | | | |  |  |
| Shoulder dystocia | | | | n (%) | | | 0 | | | | (0.0) | | | | | | | 0 | | | | | | (0.0) | | | | N/A | | | | | |  |  |
| Missing | | | | n | | | 1 | | | |  | | | | | | | 2 | | | | | |  | | | |  | | |  | | |  |  |
|  | | | |  | | |  | | | | | | | | | | |  | | | | | | | | | |  | | | | | |  |  |
| **Apgar score** | | | |  | | |  | | | | | | | | | | |  | | | | | | | | | |  | | | | | |  |  |
| < 7 at 5 minutes of age | | | | n (%) | | | 0 | | | | (0.0) | | | | | | | 0 | | | | | | (0.0) | | | | N/A | | | | | |  |  |
|  | | | |  | | |  | | | | | | | | | | |  | | | | | | | | | |  | | | | | |  |  |
| **Neonatal complications** | | | |  | | |  | | | | | | | | | | |  | | | | | | | | | |  | | | | | |  |  |
| Transient tachypnea of the newborn (TTN) | | | | n (%) | | | 1 | | | | (7.7) | | | | | | | 0 | | | | | | (0.0) | | | | N/A | | | | | |  |  |
| Respiratory distress syndrome | | | | n (%) | | | 0 | | | | (0.0) | | | | | | | 0 | | | | | | (0.0) | | | | N/A | | | | | |  |  |
| Hyaline membrane disease | | | | n (%) | | | 0 | | | | (0.0) | | | | | | | 0 | | | | | | (0.0) | | | | N/A | | | | | |  |  |
| Jaundice | | | | n (%) | | | 1 | | | | (7.7) | | | | | | | 2 | | | | | | (20.0) | | | | N/A | | | | | |  |  |
| Need for phototherapy | | | | n (%) | | | 1 | | | | (7.7) | | | | | | | 2 | | | | | | (20.0) | | | | N/A | | | | | |  |  |
| Polycythaemia | | | | n (%) | | | 0 | | | | (0.0) | | | | | | | 0 | | | | | | (0.0) | | | | N/A | | | | | |  |  |
| Fetal anomaly | | | | n (%) | | | 0 | | | | (0.0) | | | | | | | 1 | | | | | | (10.0) | | | | N/A | | | | | |  |  |
| Other | | | | n (%) | | | 2^[[28]](#footnote-28)^ | | | | (15.4) | | | | | | | 3^[[29]](#footnote-29)^ | | | | | | (33.3) | | | | RR^[[30]](#footnote-30)^ 0.46  (0.10 to 2.23) | | | | | |  |  |
| Missing | | | | n | | | 0 | | | |  | | | | | | | 2^[[31]](#footnote-31)^ | | | | | |  | | | |  | | |  | | |  |  |
|  | | | |  | | |  | | | |  | | | | | | |  | | | | | |  | | | |  | | |  | | |  |  |
|  | | | |  | | |  | | | | | | | | |  | | | | | | | | | | | |  | | | | | |  |  |
| **Birth trauma** | | | |  | | |  | | | | | | | | |  | | | | | | | | | | | |  | | | | | |  |  |
| Mild | | | | n (%) | | | 0 | | | | (0.0) | | | | | 1 | | | | | | (10.0) | | | | | | N/A | | | | | |  |  |
| Moderate or serious | | | | n (%) | | | 0 | | | | (0.0) | | | | | 0 | | | | | | (0.0) | | | | | | N/A | | | | | |  |  |
|  | | | |  | | |  | | | | | | | | |  | | | | | | | | | | | |  | | | | | |  |  |
| **Incidence of neonatal hypoglycaemia in first 2-4 hours of age^[[32]](#footnote-32)^** | | | | n (%) | | | 2 | | | | (18.2) | | | | | 0 | | | | | | (0.0) | | | | | | N/A | | | | | |  |  |
| Missing | | | | n | | | 2 | | | |  | | | | | 2 | | | | | |  | | | | | |  | | |  | | |  |  |
| Instances measured after 4 hours of age | | | | n (%) | | | 1 | | | | (7.7) | | | | | 1 | | | | | | (10.0) | | | | | |  | | |  | | |  |  |
| Any instances (total) | | | | n (%) | | | 3 | | | | (27.3) | | | | | 1 | | | | | | (11.1) | | | | | |  | | |  | | |  |  |
| Missing | | | | n | | | 2 | | | |  | | | | | 1 | | | | | |  | | | | | |  | | |  | | |  |  |
|  | | | |  | | |  | | | | | | | | |  | | | | | | | | | | | |  | | | | | |  |  |
| **Admitted to NNU** | | | | n (%) | | | 4^[[33]](#footnote-33)^ | | | | (30.8) | | | | | 1 | | | | | | (10.0) | | | | | | N/A | | | | | |  |  |
| For more than 48 hours^[[34]](#footnote-34)^ | | | | n (%) | | | 1 | | | | (25.0) | | | | | 1 | | | | | | (100.0) | | | | | |  | | |  | | |  |  |
| Admitted for hypoglycaemia^51^ | | | | n (%) | | | 1 | | | | (25.0) | | | | | 0 | | | | | | (0.0) | | | | | |  | | |  | | |  |  |
|  | | | |  | | |  | | | |  | | | | |  | | | | | |  | | | | | |  | | |  | | |  |  |

1. For outcomes with ≥ 2 in each arm only [↑](#footnote-ref-1)
2. CI denotes confidence interval [↑](#footnote-ref-2)
3. Waking/fasting test is defined as the first measurement of the day after 2am, before 12 noon and > 4 hours since the previous measurement [↑](#footnote-ref-3)
4. SD denotes standard deviation [↑](#footnote-ref-4)
5. Denotes mean difference [↑](#footnote-ref-5)
6. Post-prandial test is defined as non-fasting measures [↑](#footnote-ref-6)
7. VR denotes variance ratio, F [↑](#footnote-ref-7)
8. Denotes risk ratio [↑](#footnote-ref-8)
9. IQR denotes interquartile range (25^th^ to 75^th^ percentiles) [↑](#footnote-ref-9)
10. Denotes median difference [↑](#footnote-ref-10)
11. CI denotes confidence interval [↑](#footnote-ref-11)
12. Measured between 38 and 40 weeks’ gestation, participants were asked ‘If you were given the choice in the future, would you prefer to receive insulin injections or the glibenclamide tablets if you had diabetes again?’ [↑](#footnote-ref-12)
13. SD denotes standard deviation [↑](#footnote-ref-13)
14. MD denotes mean difference [↑](#footnote-ref-14)
15. Missing weight at 36 weeks (±1 week) of gestation [↑](#footnote-ref-15)
16. RR denotes risk ratio [↑](#footnote-ref-16)
17. Spontaneous vaginal delivery (SVD) or assisted vaginal delivery (AVD) as the reference category, compared with Caesarean section (elective or emergency). [↑](#footnote-ref-17)
18. IQR denotes interquartile range (25^th^ to 75^th^ percentiles) [↑](#footnote-ref-18)
19. Med D denotes median difference [↑](#footnote-ref-19)
20. Number of babies born to women who have delivered [↑](#footnote-ref-20)
21. For outcomes with ≥ 2 in each arm only [↑](#footnote-ref-21)
22. CI denotes confidence interval [↑](#footnote-ref-22)
23. At < 24 weeks of gestation [↑](#footnote-ref-23)
24. Stillbirths and early neonatal death (live birth, died < 7 days old) [↑](#footnote-ref-24)
25. Live birth, died between 7 and 27 days [↑](#footnote-ref-25)
26. SD denotes standard deviation [↑](#footnote-ref-26)
27. MD denotes mean difference [↑](#footnote-ref-27)
28. 1 plethoric, 1 admitted to NNU as baby was jittery [↑](#footnote-ref-28)
29. 1 plethoric, 1 IVAB given due to maternal pyrexia, 1 large cephalohaematoma [↑](#footnote-ref-29)
30. RR denotes risk ratio [↑](#footnote-ref-30)
31. 1 missing Polycythaemia, 1 missing Other [↑](#footnote-ref-31)
32. Defined as any of the following: blood glucose <2.6 mmol/l in first 2–4 hours of age, admission to neonatal unit for hypoglycaemia, or treated with intravenous glucose or any other drug to increase blood glucose [↑](#footnote-ref-32)
33. 1 admitted for hypoglycaemia, 1 for observation due to tachypnoea and jaundice, 1 because baby was jittery at 24 hours, but no low blood glucose results were recorded, 1 for non-medical reason [↑](#footnote-ref-33)
34. Denominator is the number admitted [↑](#footnote-ref-34)
